# Cyclohexa-2,5-diene-1,4-dione-based antiproliferative agents: design, synthesis, and cytotoxic evaluation

**DOI:** 10.1186/1756-9966-32-24

**Published:** 2013-04-30

**Authors:** Carmen Petronzi, Michela Festa, Antonella Peduto, Maria Castellano, Jessica Marinello, Antonio Massa, Anna Capasso, Giovanni Capranico, Annalisa La Gatta, Mario De Rosa, Michele Caraglia, Rosanna Filosa

**Affiliations:** 1 Department of Pharmaceutical and Biomedical Sciences, University of Salerno, via Ponte Don Melillo, Fisciano, SA, 84084, Italy; 2 Department of Biochemistry G. Moruzzi, University of Bologna, via Irnerio 48, Bologna, 40126, Italy; 3 Department of Chemistry, University of Salerno, via Ponte Don Melillo, Fisciano, SA, 84084, Italy; 4 Department of Biochemistry, Biophysics and General Pathology, Second University of Naples, via Costantinopoli 16, Naples, 80138, Italy

**Keywords:** Apoptosis, Cytotoxicity, Benzoquinones, Melanoma cell line M14, PARP, Reactive oxygen species

## Abstract

**Background:**

Tumors are diseases characterized by uncontrolled cell growth and, in spite of the progress of medicine over the years, continue to represent a major threat to the health, requiring new therapies. Several synthetic compounds, such as those derived from natural sources, have been identified as anticancer drugs; among these compounds quinone represent the second largest class of anticancer agents in use. Several studies have shown that these act on tumor cells through several mechanisms. An important objective of this work is to develop quinoidscompounds showing antitumor activity, but with fewer side effects. The parachinone cannabinol HU-331, is a small molecule that with its core 4-hydroxy-1,4-benzoquinone, exhibits a potent and selective cytotoxic activity on different tumor cell lines. A series of derivatives 3-hydroxy-1,4-benzochinoni were thus developed through HU-331 chemical modifications. The purpose of the work is to test the ability of the compounds to induce proliferative inhibition and study the mechanisms of cell death.

**Methods:**

The antitumor activities were evaluated *in vitro* by examining their cytotoxic effects against different human cancer cell lines. All cell lines tested were plated in 96-multiwell and treated with HU-100-V at different concentrations and cell viability was evaluated byMTT assay. Subsequently via flow cytometry (FACS) it was possible to assess apoptosis by the system of double labeling with PI and Annexin-V, and the effect of the compounds on ROS formation by measuring the dichlorofluorescein fluorescence.

**Results:**

The substitution by n-hexyl chain considerably enhanced the bioactivity of the compounds. In details, 2-hexyl-5-hydroxycyclohexa-2,5-diene-1,4-dione (V), 2,5-Dimethoxy-3-hexyl-2,5-cyclohexadiene-1,4-dione (XII) and 2-hydroxy-5-methoxy-3-hexyl-cyclohexa-2,5-diene-1,4-dione (XIII) showed most prominent cytotoxicity against almost human tumour cell lines. Compound V was further subjected to downstream apoptotic analysis, demostrating a time-dependent pro-apoptotic activity on human melanoma M14 cell line mediated by caspases activation and poly-(ADP-ribose)-polymerase (PARP) protein cleavage.

**Conclusions:**

These findings indicate that 2-hexyl-5-idrossicicloesa-2,5-diene-1,4-dione can be a promising compound for the design of a new class of antineoplastic derivatives.

Carmen Petronzi, Michela Festa, Antonella Peduto and Maria Castellano: equally contributed equally to this work.

## Introduction

Several benzoquinones have been found to be effective in the treatment of some forms of cancer; previous studies demonstrated that these drugs act on cells by numerous mechanisms, such as apoptosis, abrogation of the cell cycle, activation of caspases, stimulation of the production of reactive oxygen species (ROS), inhibition of topoisomerases I and II, activation of intracellular second messengers, and production of free radicals to attack DNA. However, their cumulative heart toxicity limits their use^1^; therefore, an important goal of present and future work is to develop quinoid compounds that display anticancer activity but with less side effects.

Among the 1,4 benzoquinones, there are several naturally occurring quinones having potent anticancer activity. A recent work [1] demonstrated that Ardisianone, a natural benzoquinone derivative, displayed anti-proliferative and apoptotic activities against human hormone-refractory prostate cancer cells (HRPC), PC-3, and DU-145. Previous investigations also showed that Primin isolated from the leaves of *Miconia Lepidota* present in Suriname forests, exhibited activity towards mutant yeast strains, indicative of their cytotoxicity and potential antitumor activity [2]. Furthermore Kaul and co-workers isolated a known cytotoxic quinine Irisoquin which demonstrated cytotoxic properties [3]. In previous reports Muhammad et al. [4] evaluated cytotoxic and antioxidant activities of alkylated benzoquinones from the leaves of *Maesa Lanceolata*. Moreover, another important 2,5-dihydroxy-3-alkyl-1,4-benzoquinone is Embelin known for a very long time in Indian and Chinese pharmacopoeias. It is the major constituent in the extracts of various parts of the shrub *Embelia Ribes*. Embelin and its derivatives possess analgesic, anti-inflammatory, antioxidant, antitumor and antifertility properties [5-7].

Important results have been described with HU-331, that exhibited potent and selective cytotoxicity against several tumorigenic cell lines such as Burkitt’s lymphoma, glioblastoma, breast, prostate and lung cancer [8]. Recent findings described that this derivative is strongly antiangiogenic at concentration as low as 300 nM by directly inducing apoptosis of vascular endothelial cells [9-11].

As a part of our research program devoted to the preparation and evaluation of new antiproliferative agents, [12-14] the para-quinone of cannabinol HU-331 (1) was selected as biologically validated starting point for compound library development, in order to evaluate the structural requirements important for biological activity and in particular the role of the substituents linked to the quinone nucleus.

We prepared compounds analogues whose structure closely resembles the natural compound, thus the 2-hydroxy 1,4 benzoquinone core was not changed.

## Methods

### Chemistry

Compounds I-V (series I) retain the same hydroxy-1,4-benzoquinone core of lead, modifications were carried out on the alkyl chain that was elongated and shifted, cycloalkenyl substituent in position 2 of HU-331 was removed (compound V) or replaced by a cyclohexyl (I and II) or by a cyclohexylmethyl moiety (III and IV).

**Figure 1:**
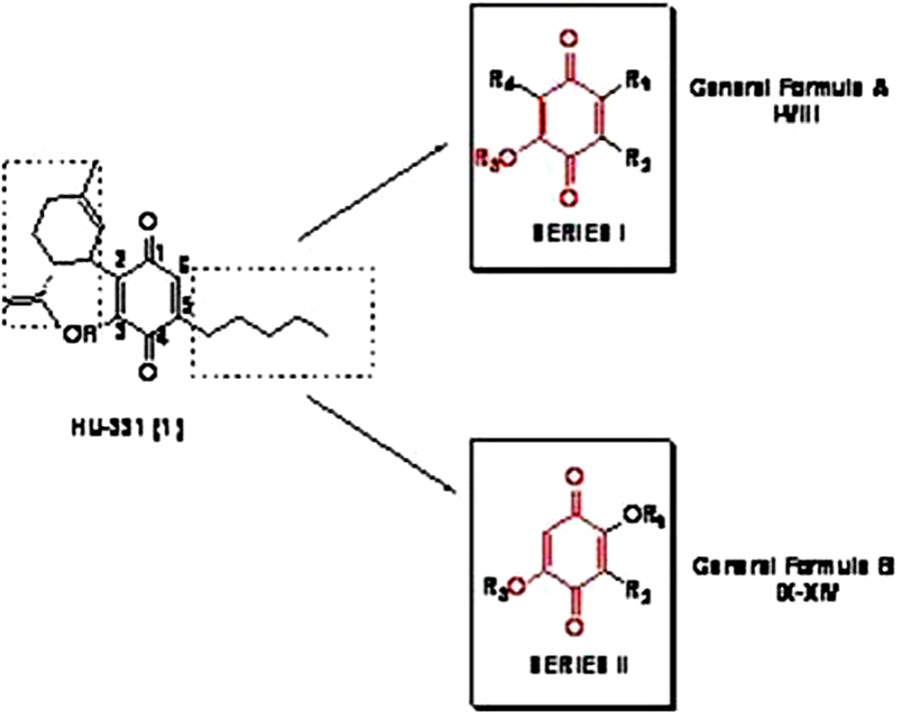
Development of compounds of general formula A and B.

On the other hand, to evaluate the influence of position of alkyl chain and hydroxy group on the 1,4-benzoquinone nucleus, compounds VI, VII and VIII were prepared. In parallel, we studied the variation of the cytotoxicity in a series of 2,5-dihydroxy-3-alkyl-1,4-benzoquinone system (series II). These compounds (IX- XIV) are characterized by a butyl or hexyl chain in position 3 of quinone ring which is 2,5 disubstituted with hydroxy or methoxy groups (Figure 1).

Compounds I and II were prepared starting from commercially available 1,3-dimethoxybenzene (2a) and 1-hexyl-2,4-dimethoxybenzene (2b) that were easily prepared according to procedure described by Kikuchi and co-workers [12-14]. Condensation of cyclohexanone with 2a-b gave the tertiary alcohols 3a-b in 70% and 80% yields respectively.

In order to remove their hydroxyl groups, 3a-b were submitted to the Barton-McCombie procedure, an extremely useful method with widespread application in synthetic organic chemistry. Compounds obtained were oxidized into quinoid compounds I (65% yield) and II (60% yield). Deprotection and final oxidation in air under basic conditions, led to the formation of the desired quinones III and IV in 55% and 60% yield, respectively.

The choice of the effective oxidizing agent and conditions used in the last step of both the synthetic schemes, derived from a set of experiments applied to the oxidation of the commercially available model compound 4-hexylresorcinol 10 to V, as shown in Table 1.

**Table 1:** Oxidation of 4-Hexylresorcinol to V with different oxidizing agents

**Entry**	**Conditions**	**Yield (%)**
**1**	Salcomine (0.01 eq.) ,DMF, 110°C, 3 h	30^a^
**2**	Salcomine (1 eq.), DMF, rt, 6 h	50^a^
**3**	Etanol/air, Petroleum ether, KOH 5%, 0°C, 5 h	33
**4**	Fremy’s salt, Aliquat336/Ph, Na_2_CO_3,_ rt, 18 h	30^a^
**5**	Fremy’s salt, H_2_O, Na_2_HPO_4,_ rt to 50°C, 36 h	22
**6**	Fremy’s salt, H_2_O/EtOAc, Na_2_HPO_4,_ 0°C to rt, 18 h	50
**7**	Fremy’s salt, H_2_O/THF, Na_2_CO_3,_rt, 10 h	60

^a^Conversion of starting material in o-hydroxyquinone.

For example the conversion of 10 to V was tried in the presence of Salcomine, a coordination complex derived from the salen ligand and cobalt.

This catalyst is known to catalyze the oxidation of 2,6-disubstituted phenols by dioxygen but in our case a complete conversion of the starting material 10 in *o*-hydroxyquinone was observed (Entry 1–2).

In another attempt, using a catalytic amount of ethanol in air, a solution of 5% of KOH as base and petroleum ether as solvent (Entry 3), only a little quantity of starting material was converted into quinone V. For these reasons Fremy’s salt (disodium nitrosodisulfonate) was tried as oxidizing agent, under several conditions (Entry 4–7). However, in the presence of Na_2_CO_3_ as base and a mixture of H_2_O/THF as solvent, was obtained the best results (Entry 7) [15].

Ag(I)-promoted Suzuki–Miyaura cross-coupling of 1-bromo-2,3.5-trimethoxybenzene (11) and hexylboronic acid pinacol ester furnished intermediate 12 in 20% yield. Deprotection and simultaneous oxidation to 2-hydroxy-p-benzoquinone (VI) was achieved treating 12 with an excess of BBr_3._ Oxidation with ammonium cerium nitrate (CAN) in a mixture of acetonitrile and water allowed to obtain 2-methoxy derivative VII. Compound VIII was synthesized starting from 1,3-dimethoxybenzene (2a) which was subjected to a ortho-metalation reaction in presence of n-BuLi. Coupling reaction of 1,2,4,5-tetramethoxybenzene with two different alkyl iodides, in presence of n-BuLi and hexamethylphosphoramide (HMPA) furnished intermediates 15 and 16 in moderate yields (42% and 37% respectively). CAN-mediated oxidative reaction provided^22^a mixture of 2,5-dimethoxy (IX and XII) and 2-hydroxy-5-methoxy derivatives (X and XIII)

Treatment of IX and XII with 2M NaOH allowed to obtain 2,5-dihydroxy derivatives XI and XIV in good yields (72% and 89%).

### General procedures

All reagents were analytical grade and purchased from Sigma-Aldrich (Milano-Italy). Flash chromatography was performed on Carlo Erba silica gel 60 (230÷400 mesh; Carlo Erba, Milan, Italy). TLC was carried out using plates coated with silica gel 60F 254 nm purchased from Merck (Darmstadt, Germany). Melting points were determined in open capillary tubes on a Electrothermal 9100. ^1^H and ^13^C NMR spectra were registered on a Bruker AC 300. Chemical shifts are reported in ppm. All target compounds were assessed for purity by analytical high performance liquid chromatography (HPLC), using an Agilent 1100 series, UV detector monitoring at 254 nm, HP Chem Station software, and a Waters C18 RP-column (5 μm, 300 mm × 3.9 mm). All target compounds were found to be >95% purity. MS spectrometry analysis ESI-MS was carried out on a Finnigan LCQ Deca*ion trap* instrument*.* Microanalyses were carried out on Carlo Erba 1106 elemental analyzer.

### Biological studies

#### Cell culture

Our experimental models consist of several cell lines derived from human cancers of different histogenesis. The cells were grown in RPMI or DMEM supplemented with heat inactivated 10% FBS, 20 mM HEPES, 100 U/ml penicillin, 100 μg/ml streptomycin, 1% L-glutamine in a humidified atmosphere of 95% air/5% CO_2_ at 37°C [16].

Analysis of cell proliferation was performed in the presence of all derivatives on all cell lines seeded in 96-well plates at the different densities depending on the cell type. Pancreas cancer cell lines ( BXPC3, PANC-1) were plated to the average density of 3,600 cells/ well. Prostate cancer cell lines (DU145, PC3, LNCAP) were plated to the average density of 2,000 cells/ well. Melanoma cell lines (COLO38, A375, M14) were plated to the average density of 1,800 cells/ well. Renal cancer cell lines (A498, RXF393, SN12C, 769P) and glioblastoma cell lines ( LN229, U87 MG, U373 MG) were plated to the average density of 1,900 cells/ well. Breast cancer cell lines (CG5, MCF-7, MDA-MB 231, MDA-MB 468, MDA-MB 436 ) were plated to the average density of 3,100 cells/ well. After 24 h incubation at 37°C, the cells were treated with increasing concentrations of compounds (0,037-50 μM). Cells were incubated under these conditions for 72 h.

### MTT bioassay

Human cancer cells (3 × 10^3^) were plated in 96-well culture plates in 90 μL of culture medium and incubated at 37°C in humidified atmosphere of 5% CO_2_. The day after, 10 μL aliquot of serial dilutions of compounds (1–50 μM) was added to the cells and incubated for 72 h. The cell viability was assessed with MTT [3-(4,5-dimethylthiazol-2-yl)-2,5-diphenyl tetrazolium bromide] method [17]. After 72 h of treatment with derivatives MTT solution 5 mg/ml in PBS was added to each well. The plates were then incubated at 37°C for an additional 4 h to allow MTT to form formazan crystals by reacting with metabolically active cells. The formazan crystals were solubilized in a 1N isopropanol/HCl 10% solution at 37°C, on a shaking table for 20 min. The absorbance values of the solution in each well were measured at 570 nm using a micro plate reader.

Cell viability was determined by the formula:as previously reported [18]. All MTT experiments were performed in quadruplicated and repeated at least three times. Data are as mean ± standard deviation (SD). Each IC_50_ mean value was obtained from four independent experiments.

$$\mathrm{cell}\;\text{viability}\;\left(\%\right)=\frac{\left(\text{absorbance of the treated wells absorbance of the blank control wells}\right)}{\left(\text{absorbance of the negative control wells absorbance of the blank control wells}\right)}\;\times \;100\%$$

### Apoptotic cell death

Hypodiploid DNA was analysed using the method of propidium iodide (PI) staining and flow cytometry as described [19]. At the end of incubation with HU compounds, with or without pretreatment with pan-caspase inhibitor Z-vad-fmk, purchased from BD Pharmingen (BD Bioscience, Bedford, USA), cells were washed in phosphate-buffered saline (PBS) and resuspended in 500 μL of a solution containing 0.1% sodium citrate, 0.1% Triton X-100 and 50 μg/ml propidium iodide (Sigma-Aldrich, Italy). After incubation at 4°C for 30 minutes in the dark, cell nuclei were analyzed with Becton Dickinson FACScan flow cytometer using the Cells Quest program. Cellular debris was excluded from analysis by raising the forward scatter threshold, and the DNA content of the nuclei was registered on logarithmic scale. The percentage of the cells in the hypodiploid region was calculated [20].

### Western blotting analysis

Total intracellular proteins were extracted from the cells by membrane disruption in lysis buffer 50 mM Tris-HCl, 1% Na-deoxycholate, 1% SDS and 0.5% IGEPAL (All from Sigma-Aldrich, Gallarate, Italy) containing protease and phosphatase inhibitors (1mM PMSF, 1 μg/ml leupeptin, 1μg/mL pepstatin, 1μg/mL aprotinin, 1 μM Na_3_PO_4_, 1 μM NaF; all from Sigma Sigma-Aldrich, Gallarate, Italy) on ice for 20 min. The cell lysate was then centrifugated at 10,000 × g at 4°C for 15 min. The supernatant was collected as protein extract. Protein content was estimated according to Biorad protein assay (BIO-RAD, Milan, Italy) and the samples either analysed immediately or stored at −80°C. Total protein (30 μg) samples were loaded into a 10-12% acrylamide gels and separated by SDS-PAGE in denaturating conditions at 150 V.

The separated proteins were then transferred electrophoretically (100 mA per blot 90 min; Trans Blot Semi-Dry, BIO-RAD) to nitrocellulose paper (Immobilon-NC, Millipore, Bedford, USA) soaked in transfer buffer (25 mM Tris, 192 mM glycine, Sigma-Aldrich) and 20% methanol vol/vol (Carlo Erba, Milan, Italy) [21]. Non specific binding was blocked by incubation of the blots in 5% no fat dry-milk powder (BIO-RAD) in TBS/0.1%Tween (25 mM Tris; 150 mM NaCl; 0.1% Tween vol/vol, Sigma-Aldrich) for 60 min. After washing, the blots were incubated overnight at 4°C with the following primary antibodies: mouse monoclonal anti-PARP? (diluted 1:1,000) and anti-XIAP (all from Santa-Cruz Biotechnology, Santa Cruz,CA). After incubation with the primary antibodies and washing in TBS/0.1% Tween, the appropriate secondary antibody, either anti-mouse (diluted 1:5,000), or anti-rabbit (diluted 1:5,000) (both from Sigma-Aldrich, Italy) was added for 1h at room temperature. Immunoreactive protein bands were detected by chemiluminescence using enhanced chemiluminescence reagents (ECL) and exposed to Hyperfilm (both from Amersham Biosciences, Italy). The blots were then scanned and analysed (Gel-Doc 2000, BIO-RAD).

### Flow cytometric estimation of intracellular redox state

The effect of all compounds on intracellular reactive oxygen species (ROS) was evaluated by measuring dichlorofluorescein (DCF) fluorescence [22]. Cells were incubated in presence and absence of compounds. At the end of incubation time, cells were washed and resuspended (2 × 105 cells/ml) in Hank’s balanced salt solution (HBSS) cointaining 10 μM 2′,7′-dichlorodihydrofluorescein diacetate (DCFH-DA). Following a further 20 min incubation at 37°C, DCF fluorescence was monitored by flow cytometry (FL1-H channel). In order to estimate the antioxidant potential of the compounds, control and teatred cells were exposed to 300 μM of the oxidant tert-bytylhydroperoxide (t-BOOH) for 30 min at 37°C before DCFH-DA loading.

### Topoisomerase I-Mediated DNA cleavage reactions

Human recombinant Top1 was purified from Baculovirus as previously described [23]. DNA cleavage reactions were performed using a 22-bp DNA oligonucleotide with a prominent Topoisomerase I cleavage site. Single-stranded oligonucleotide was labeled according to the manufacturers’ instructions by using terminal deoxynucleotidyltransferase (USB Corporation, Cleveland, OHIO) that adds a single labeled cordycepin molecule (γ-32P, 5000 Ci/mmol, PerkinElmer Life and Analytical Sciences, MA) to the 3′ terminus. Unincorporated nucleotides were removed by QIAquick Nucleotide Removal Kit (Qiagen, Hilden, Germany). The duplex DNA oligonucleotide was annealed by addition of an equal concentration of the complementary strand, heated to 95°C and slow cooled to room temperature. For the Toposomerase I cleavage reaction, DNA oligonucleotides were reacted for 20 min at 25°C with a 12 ng/mL solution of human Topoisomerase I and the desired amount of drugs, in 10 mM Tris–HCl pH 7.5, 50 mM KCl, 5 mM MgCl2, 0.1 mM EDTA and 15 μg/mL bovine serum albumin. Reactions were stopped by adding 0.5% SDS and formamide containing 0.25% bromophenol blue and xylene cyanol, heated at 95°C for 5 min and chilled on ice. Reaction products were separated in 20% polyacrylamide denaturing sequencing gels. Dried gels were visualized using a B40 Storm phosphor imager (Amersham Biosciences, GE Healthcare, UK).

### Topoisomerase II-Mediated DNA cleavage reactions

DNA was purchased from Invitrogen Corporation (Carlsbad, CA). It represents a portion of SV40 sequence, in particular from position 3449 to 3538, that contains prominent topoisomerase II cleavage sites [24]. DNA was purified on denaturing 20% polyacrylamide gel, recovered by soaking gel slices in water and then ethanol precipitated. Single-stranded DNA was 5′-labeled using T4 polynucleotide kinase (New England Biolabs, Ipswich, MA) with [γ-32P]ATP (3000 μCi/mmol, PerkinElmer Life and Analytical Sciences, MA) according to the manufacturers’ instructions. Unincorporated nucleotides were removed by QIAquick Nucleotide Removal Kit (Qiagen, Hilden, Germany). The duplex DNA was annealed by addition of an equal concentration of the complementary strand, heating to 95°C and slow cooling to room temperature. For the topoisomerase II cleavage reaction, DNA fragments were reacted for 30 min at 37°C with 30 units/sample of human topoisomerase II, purified as previously described, [25] and the desired amount of drugs, in 40 mM Tris–HCl, pH 7.5, 80 mMKCl, 10 mM MgCl_2_, 5 mM DTT, 1 mM ATP, and 15 μg/mL bovine serum albumin. Reactions were stopped by adding 1% SDS and 0.2 mg/mL proteinase K and incubated at 42°C for 45 minutes. Samples were then ethanol precipitated, resuspended in 10 μL of formamide containing 0.25% bromophenol blue and xylene cyanol, heated at 95°C for minutes and chilled on ice. Reaction products were separated in 20% polyacrylamide denaturing sequencing gels. Dried gels were visualized using a B40 Storm phosphor imager (Amersham Biosciences, GE Healthcare, UK).

### Statistical analysis

All results are shown as mean ± SEM of three experiments performed in triplicate. The optical density of the protein bands detected by Western blotting was normalized on β-actin levels. Statistical comparison between groups were made using ANOVA followed by Bonferroni parametric test. Differences were considered significant if P < 0.05.

## Results and discussion

### Biological evaluation

For the evaluation of cytotoxicity, five different human cancer cell lines were utilized: M14 and A375 (melanoma cell lines), MCF-7 (human breast cancer cell), PC3 (human prostatecancer cell line), A498 (human renal carcer cell line). The survival percentage was determined after a period of 72 h at screening concentrations from 50 to 1 μM, using the survival percentage obtained from the cells treated only with the solvent (DMSO at 0.5%) as a reference. Our experiments confirmed the cytotoxic activity of HU-331. Most of the compounds displayed moderate cytotoxicity against cancer cell lines in relatively lower micromolar concentrations when compared to the standard. Among the compounds, derivatives V, IX, XII and XIII showed significant cytotoxicity in most of the cell lines, displaying similar or slightly weaker potency than positive control. Compound V can be considered the most interesting compound that showed a good anticancer activity against all tumor cell lines and was more potent than HU-331 against M14 (7 μM *vs* 15 μM). The structure-activity relationship studies regarding the first series of compounds revealed that the n-hexyl chain in position 5 of the hydroxy-quinone ring was fundamental for the anticancer activity (compounds II, IV and V), in fact compounds I and III, which lacked of the alkyl chain, were completely inactive. At the same time, the change of position of alkyl chain was clearly detrimental (VI, VII and VIII *vs* V). No relevant influence on the activity was observed if a methylene spacer was inserted between cyclohexyl and 1,4-benzoquinone ring (IV *vs* II). As concern for compounds of series II, the 5-methoxy-1,4-benzoquinone derivatives IX,XII and XIII were the most active compounds of the series, while compound X was slightly active only against M14 cell line. Considering the importance of the length of the side chain, the hexyl chain seemed to be more favorable for the activity but further investigations will be done. 2,5-dihydroxy-1,4-benzoquinone derivatives XI and XIV were completely inactive against all tumor cell lines at the concentration of 100 μM. Structure-activity relationship evaluations, comparing compounds of first and second series, demonstrated that the introduction of a methoxy (XII) or hydroxy (XIII) group on the 1,4-benzoquinone ring of compound VII caused a strong improvement in the cytotoxicity against almost tumor cell lines, except A498. On the contrary, if another hydroxy group was inserted on the quinone core of compound VI, no improvement of activity was recorded (compound XIV). Having identified, from the first screening, the most cytotoxic compound against all tumor cell lines, we have carried out a screening on other solid tumor cell lines to confirm the cytotoxic activity of this molecule. Moreover, we investigated the molecular mechanisms underlying the antiproliferative activity in comparison with the natural compound HU-331 on M14 cells. However all data are reported in Table 2. The MTT viability assay showed that compound V has good antiproliferative properties against all tested solid human cancer cell lines (Table 3).

**Table 2:** Effects of HU compounds on proliferation of several cancer cell lines

					**Cell lines IC**_**50**_**[μM]**				
**Cpd**	**R**_**1**_	**R**_**2**_	**R**_**3**_	**R**_**4**_	**M14**	**MCF-7**	**PC3**	**A498**	**A375**
**I**	H	H	H		>100	>100	>100	>100	>100
**II**	n-hexyl	H	H		23 ± 0.12	28.13 ± 0.07	41 ± 0.20	34.91 ± 3.82	>100
**III**	H	H	H		>100	>100	>100	>100	>100
**IV**	n-hexyl	H	H		45.6 ± 0.20	37.3 ± 0.34	38 ± 0.12	**28.8 ± 0.04**	30.7 ± 0.12
**V**	n-hexyl	H	H	H	**7.0 ± 0.10**	**18.7 ± 0.06**	**24.3 ± 0.20**	**19.8 ± 0.02**	**12.9 ± 0.06**
**VI**	H	n-hexyl	H	H	-	>100	>100	>100	>100
**VII**	H	n-hexyl	CH_3_	H	-	>100	>100	>100	>100
**VIII**	H	H	CH_3_	n-hexyl	-	>100	>100	>100	>100
**IX**	-CH_3_	n-butyl	CH_3_	H	24.5 ± 0.15	**12 ± 0.03**	**17.9 ± 0.20**	51 ± 0.02	**17.6 ± 0.05**
**X**	H	n-butyl	CH_3_	H	35 ± 0.64	>100	>100	>100	>100
**XI**	H	n-butyl	H	H	>100	>100	>100	>100	>100
**XII**	-CH_3_	n-hexyl	CH_3_	H	**10.7 ± 0.15**	**16.2 ± 0.03**	**18.8 ± 0.03**	>100	**21.0 ± 0.04**
**XIII**	H	n-hexyl	CH_3_	H	**14.1 ± 0.15**	**13.9 ± 0.04**	**20.1 ± 0.20**	>100	**18.1 ± 0.04**
**XIV**	H	n-hexyl	H	H	>100	>100	>100	>100	>100
**H331**					15.0 ± 0.09	24.5 ± 0.15	32.0 ± 0.15	34.6 ± 0.23	21.8 ± 0.03

Cell viability was assessed through MTT assay. Data represent the mean ± SD values of three independent determinations performed in triplicate. ***A375***, ***M14***, human melanoma cells; ***MCF-7***, human breast cancer cells; ***PC3***, Human prostate cancer cell line, ***A498***, Human renal cancer cell line.

**Table 3:** Cytotoxic activity of compound V in solid human cancer cell lines

**Cell lines**		**IC**_**50**_**(μM)**
Prostate	LN-CAP	**15.2**
	DU-145	**19.2**
Pancreas	BX-PC3	**19.8**
	PANC-1	31.6
Renal	SN12C	23.6
	RXF393	**19.9**
	769P	34.6
Glioblastoma	LN229	**18.2**
	U373MG	23.6
	U87MG	30.8
Breast	CG-5	34.6
	MDA-MB 231	33.6
	MDA-MB 468	41.2
	MDA-MB 436	40.1

### In vitro cytotoxicity

**Figure 2:**
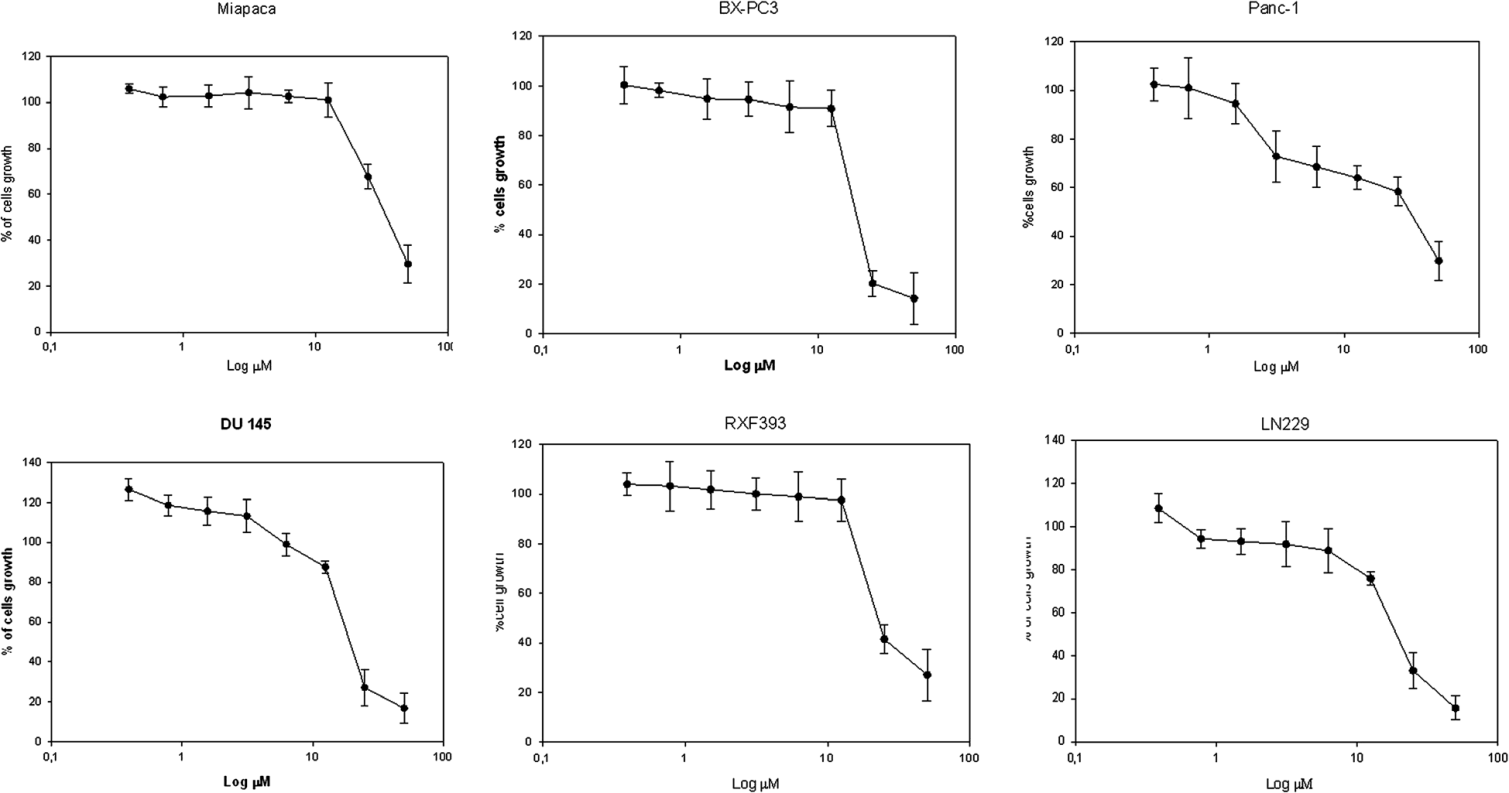
Dose–response curves from the treatment of different cell lines with the molecule HU-100-V with an IC50 between more less than 20 μM.

The cytotoxicity of HU-100-V was evaluated on different cell lines derived from different tumors. In all treatments the cells were plated in 96-multiwell in the presence of increasing concentrations of the drug, (0,37 to 50 μM). The MTT viability assay showed that HU 100-V decreased the viability of most of the cell lines tested in a time- and dose-dependent manner for which they are achieved good values of IC50 (concentration inhibiting 50% of growth). Especially, prostate cancer DU-145, pancreas cancer BX-PC3 [26,27], renal cancer RXF393 and glioblastoma cancer LN229 cells have proved to be the most sensitive to this treatment, with IC_50_ values of less than 20 micromolar (Table 3 and Figure 2).

### Apoptotic cell death

**Figure 3:**
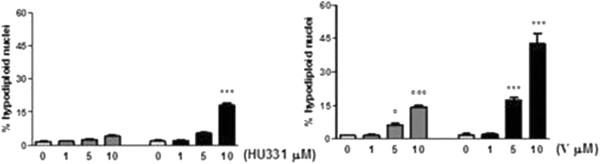
**Effects of HU compounds on apoptosis of human melanoma M14 cells.** Analysis of the % of apoptotic cells was performed using PI cell permeabilization staining. M14 cells were treated with different concentrations of HU-331 and V (1–10 μM) for 24–72 h. Cells were then collected and % of hypodiploid nuclei was analyzed by flow cytometry (*** P < 0.001 vs 72 h control cells; ° P < 0.05, °°° P < 0.001 vs 24 h control cells). Results are expressed as mean ± SEM of three experiments performed in triplicate.

To ascertain whether loss of cell viability was mediated by effects on apoptosis we directly analyzed the effects of either V or HU-331 on apoptosis of M14 cells by using PI-staining of DNA fragmentation after cell permeabilization. Cells were treated with different concentrations (1–10 μM) of V and HU-331 for 24 and 72 hours and then the population of sub-G1 cells (hyplodiploid nuclei) was determined. Compound V induced apoptosis of M14 cells in a concentration-dependent manner with 40% of cell death at 10 μM after 72 h, whereas a small pro-apoptotic effect was observed with 10 μM HU-331 (Figure 3). These results showed that the cytotoxic effect of V is dependent by an apoptotic mechanism that is more significant than HU-331 effect on M14 cells.

### Caspases involvement

**Figure 4:**
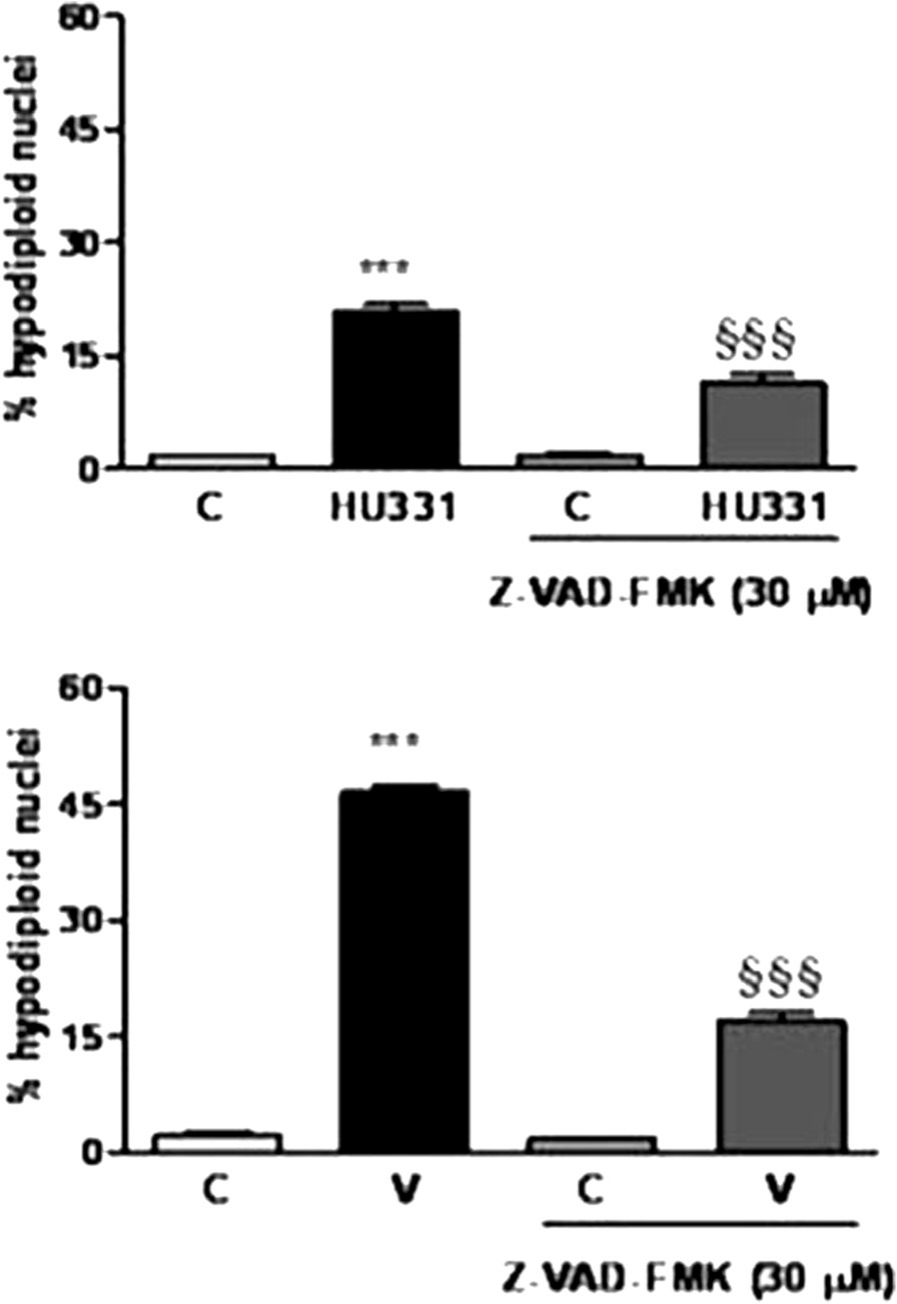
**Effects of the caspase inhibitor Z-VAD-FMK on apoptosis induced by HU331 and V in human melanoma M14 cells.** Z-VAD-FMK (30 μM) was administered 30 min before incubation with HU-331 and V (10 μM) for 72 h and the % of apoptotic cell was evaluated by flow cytometry (mean ± SEM of three experiment performed in triplicate; ***P < 0.001 vs control cells, §§§ P < 0.001 HU331 vs V treated cells.

To investigate the involvement of caspases in the mechanism of apoptosis induced by compounds, we pretreated the cells with a pan-caspase inhibitor Z-VAD-fmk for 30 min before to add V and HU-331. Results in Figure 4 show that apoptosis induced by V in presence of the inhibitor was significantly reduced indicating the involvement of caspases in the apoptotic mechanism in M14 cells.

### Cell cycle analyses

The cell cycle is divided into four phases, i.e. sub-G1, G1, S and G2. The sub-G1 represents the apoptotic cells whereas the G1 represents the first phase of cell cycle in which cell prepares itself for DNA synthesis. The cell cycle assay was performed through tagging the DNA with the PI dye as explained in “Materials and Methods”. M14 cells were plated in 6-well tissue culture plates. The cells were induced with compound V (10 μM) and the standard HU-331 (10 μM) and analyzed on a FACScan instrument using CELLQuestPRO software after time intervals of 24 h and 72 h. Cell cycle phases were compared in treated and untreated samples. No effect for either HU-331 or V was observed on cell cycle distribution of melanoma cells (data not shown).

### Intracellular pathway involvement

**Figure 5:**
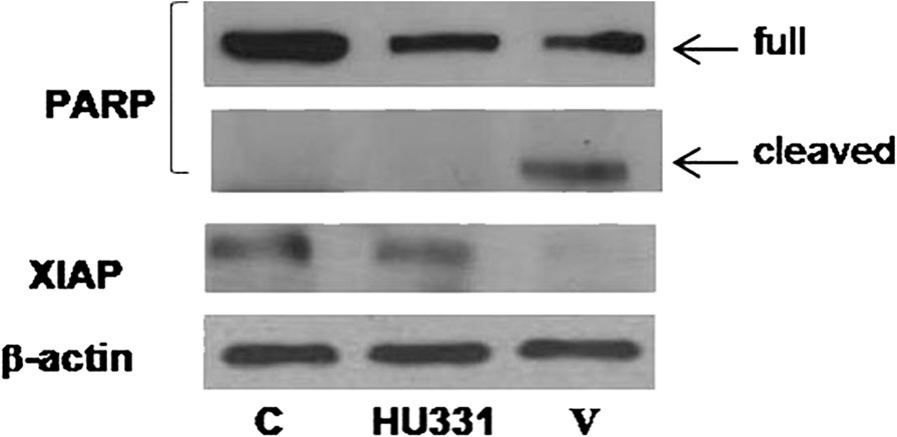
**Effect of HU compounds on intracellular ROS generation at early time points in M14 cells.** Cells were treated with V and HU331 for 30 min and then intensity of fluorescence of positive cells to DCFH-DA was analyzed by flow cytometry (FL-1channel). Results are representative of three experiments performed in triplicate. MFI:mean fluorescence intensity.

**Figure 6:**
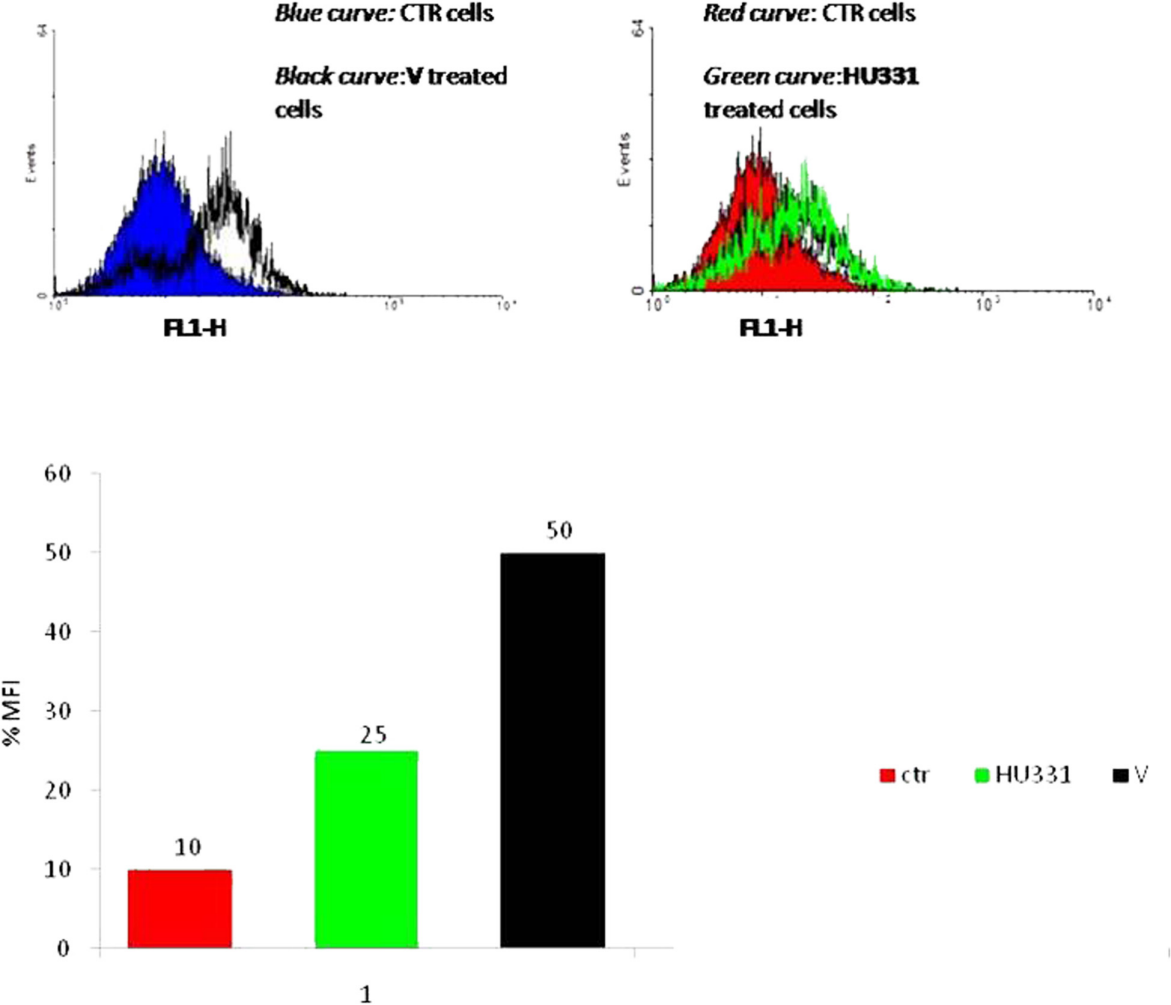
**Western blotting analysis of PARP cleavage and XIAP protein expression after incubation with HU-331 and V(10 μM) for 24 hours.** Blots are representative of three different experiments.

Evasion from apoptosis is one of the hallmarks of human cancers contributing to tumor formation and treatment resistance. The alterations in apoptosis signaling pathway often occur in drug-resistant cancer cells. In particular, defective apoptosis signaling may be caused by an increase in content of anti-apoptotic molecules and/or by a decreased content or impaired function of pro-apoptotic proteins. Thus, identification of novel substances for overcoming the drug resistance has gained much attention in cancer therapy. The drug resistance of cancer cells is a complex phenomenon comprising different intracellular processes. It was described for doxorubicin that short-term-treated CEM cells gradually developed drug resistance. In particular, caspases activation, and XIAP and PARP cleavage were blocked. Thereafter, we evaluated the effect of the active apoptotic concentrations on expression of X-linked Inhibitor of Apoptosis Protein (XIAP) and Poly (ADP-ribose) polymerase (PARP) proteins. Cells were treated with V and HU-331 at 10 μM for 24 h and then the expression of XIAP and cleavage of PARP were analyzed by western blotting. Results in Figure 5 show that apoptotic effect of V was due to PARP cleavage that leads to inactivation of this protein, importantly involved in DNA repair. No effect on PARP cleavage was observed with HU-331 treatment. We also showed (Figure 6) that V was able to abolish XIAP protein levels whereas a little effect was observed in reduction of XIAP expression after HU-331 treatment.

### ROS involvement

The quinoid anticancer agents undergo enzymatic reduction via one or two electrons to give the corresponding semiquinone radical or hydroquinone. Under aerobic conditions the semiquinone radical anion can give its extra electron to molecular oxygen to give the parent quinone and superoxide radical anion. This reaction sequence, is known as redox-cycling, and it continues until the system becomes anaerobic. Both the semiquinone and the superoxide radical anion can generate the hydroxyl radical, which is the cause of DNA strand breaks [28]. HU-331 induced cell death of human cancer cell lines is not mediated by reactive oxygen intermediates/species, as exposure to HU-331 failed to elicit the generation of reactive oxygen species. To assess the involvement of free radicals in V- mediated cell death we measured the production of reactive oxygen species (ROS) after exposure of compound at different times (5–120 min) by FACS analysis of DCFH-DA fluorescence intensity. Treatment with V increased intracellular ROS levels at early time point after 5 minutes of treatment with maximal effect after 30 min (Figure 5A), while we can confirm that the effect of HU-331 was very poor on ROS intracellular production (Figure 5B).

### Topoisomerase inhibition

To determine topoI catalytic activity, assays were carried out with supercoiled pBR322 DNA as the substrate according to protocol. Camptothecin (CPT) was the reference compound for Top1-mediated DNA cleavage reactions. Test derivatives did not increase DNA cleavage levels. Similar results were obtained with Top2. Mitonafide was the reference compound for Top2-mediated DNA cleavage reactions. The findings show that none of the assessed compounds are poisons of human Top2, thus their cellular effects can likely be due to a molecular mechanism different from topoisomerase poisoning (data not shown).

## Conclusion

Natural benzoquinone compounds are a rich source for modern, molecular targeted-specific drug discovery [29]. Over the years, a great amount of efforts have been spent to isolate individual compounds and screen for anti-cancer activity. Previous research has demonstrated that HU-331 in vivo was more active and less toxic than doxorubicin [10,11] and thus represents a promising lead compound for designing a new class of anti-cancer treatments. The aim of this study was to check the cytotoxicity of novel synthetic 1,4 benzoquinone compounds. The new derivatives together with the natural lead were tested for their anti-proliferative activity against five cancer cell lines. The general trend on which the design of these structure is based has proven to be valid in obtaining new interesting compounds. In particular, 2-hexyl-5-hydroxycyclohexa-2,5-diene-1,4-dione (V) resulted the best synthesized compound; therefore, it was further subjected to downstream apoptotic analysis. Our study demonstrated a time-dependent pro-apoptotic activity of compound V. We determined that cell death of M14 induced by V is mediated by caspases activation and poly-(ADP-ribose)-polymerase (PARP) protein cleavage. In addition we showed that HU-331 does not elicit the production of ROS while apoptosis induced by compound V could be activated by production of ROS observed after 30 min of treatment in M14 cells. We can suppose that the quinone structure permits to this compound to act as electron acceptor of free radicals that may kill cancer cells. To better define the possible mechanism of action of compounds, we also examined their dose-dependent effect on topoisomerases, as HU-331 has been proposed to be a catalytic inhibitor of topoisomerase II. We tested their ability to directly inhibit topoisomerases in cleavage assays demonstrating that our derivatives are not able to poison the nuclear enzymes. To conclude, the analyses of the present study have revealed that the synthesized quinine V has the potential to induce apoptosis in M14 cancer cell line in vitro and it is very important to note that this compound additionally has the ability to inhibit the expression of the antiapoptotic protein XIAP, a regulatory protein that suppresses apoptosis cell death by binding the caspase proteins [30,31]. On the light of interesting pharmacological results, a more extensive medicinal chemistry program has been engaged to consolidate the series and identify lead candidates for the design of more potent antitumor agents based on 2-hydroxyquinone skeleton which in turn should afford a better understanding of biological mechanisms regulating apoptosis.

## Abbreviations

ROS: Reactive oxygen species; HRPC: Hormone-refractory prostate cancer cells; HMPA: Hexamethylphosphoramide; XIAP: X-linked inhibitor of apoptosis protein; PARP: Poly (ADP-ribose) polymerase; CPT: Camptothecin; DCF: Dichlorofluorescein.

## Competing interests

The authors declare that they have no competing interests.

## Authors’ contributions

RF and MC carried out the design of the experiments and drafted the manuscript. CP, MF, AP and MC participated in the experiments of cell culture and molecular biology. JM, AM, AG and GC, participated in statistical analysis and interpretation. ALG and MDR participated in the design of the experiments. All authors read and approved the final manuscript.

## References

[B1] Yu CC Wu PJ Hsu JL Ho YF Hsu LC Chang YJ Chang HS Chen IS Guh JH Ardisianone, a natural benzoquinone, efficiently induces apoptosis in human hormone-refractory prostate cancers through mitochondrial damage stress and survivin downregulation Prostate 2013 73 2 133 145 Epub 2012 Jun 5.2012 10.1002/pros.22548 22674285

[B2] Gunatilaka AA Berger JM Evans R Miller JS Wisse JH Neddermann KM Bursuker I Kingston DG Isolation, synthesis, and structure-activity relationships of bioactive benzoquinones from Miconia lepidota from the Suriname rainforest Nat 2001 64 2 5 10.1021/np000219r 11170656

[B3] Mahmood U Kaul VK Jirovetz L Alkylated benzoquinones from Iris kumaonensis Phytochemistry 2002 61 923 926 10.1016/S0031-9422(02)00474-0 12453518

[B4] Muhammad I Takamatsu S Walker LA Mossa JS Fong HH El-Feraly FS Cytotoxic and antioxidant activities of alkylated benzoquinones from Maesa lanceolata Phytother Res 2003 17 887 891 10.1002/ptr.1237 13680818

[B5] Chitra M Sukumar E Suja V Devi CS Antitumor, anti-inflammatory and analgesic property of embelin, a plant product Chemotherapy 1994 40 109 113 10.1159/000239181 7510605

[B6] Hu R Zhu K Li Y Yao K Zhang R Wang H Embelin induces apoptosis through down-regulation of XIAP in human leukemia cells Med Oncol 2011 28 8 1584 20625944 10.1007/s12032-010-9601-5

[B7] Nikolovska-Coleska Z Xu L Hu Z Tomita Y Li P Roller PP Wang R Fang X Guo R Zhang M Lippman ME Yang D Wang S Discovery of embelin as a cell-permeable, small-molecular weight inhibitor of XIAP through structure-based computational screening of a traditional herbal medicine three-dimensional structure database Med Chem 2004 47 2430 2440 10.1021/jm030420+ 15115387

[B8] Kogan NM Rabinowitz R Levi P Gibson P Sandor D Schlesinger M Synthesis and antitumor activity of quinonoid derivatives of cannabinoids Med Chem 2004 47 3800 3806 10.1021/jm040042o 15239658

[B9] Kogan NM Blázquez C Álvarez L Gallily R Schlesinger M Guzmán M Mechoulam R A cannabinoid quinone inhibits angiogenesis by targeting vascular endothelial cells Mol Pharm 2006 70 51 59 10.1124/mol.105.021089 16571653

[B10] Kogan NM Schlesinger M Priel E Rabinowitz R Berenshtein E Chevion M Mechoulam R HU-331, a novel cannabinoid-based anticancer topoisomerase II inhibitor Mol Cancer Ther 2007 6 6173 6183 10.1158/1535-7163.MCT-06-0039 17237277

[B11] Kogan NM Schlesinger M Peters M Marincheva G Beeri R Mechoulam R A cannabinoid anticancer quinone, HU-331, is more potent and less cardiotoxic than doxorubicin: a comparative in vivo study JPET 2007 322 646 653 10.1124/jpet.107.120865 17478614

[B12] Filosa R Peduto A De Caprariis P Saturnino C Festa M Petrella A Pau A Pinna GA La Colla P Busonera B Loddo R Synthesis and antiproliferative properties of N3/8-disubstituted 3,8-diazabicyclo[3.2.1]octane analogues of 3,8-bis[2-(3,4,5-trimethoxyphenyl)pyridin-4-yl]methyl-piperazine MedChem 2007 42 293 306 10.1016/j.ejmech.2006.11.013 17254669

[B13] Filosa R Peduto A Micco SD Caprariis P Festa M Petrella A Capranico G Bifulco G Molecular modelling studies, synthesis and biological activity of a series of novel bisnaphthalimides and their development as new DNA topoisomerase II inhibitors MedChem 2009 17 13 24 10.1016/j.bmc.2008.11.024 19058969

[B14] Peduto A Pagano B Petronzi C Massa A Esposito V Virgilio A Paduano F Trapasso F Fiorito F Florio S Giancola G Filosa R Design, synthesis, biophysical and biological studies of trisubstituted naphthalimides as G-quadruplex ligands BioorgMedChem 2011 21 6419 6429 10.1016/j.bmc.2011.08.062 21944546

[B15] Petronzi C Filosa R Peduto A Monti MC Margarucci L Massa A Structure-based design, synthesis and preliminary anti-inflammatory activity of bolinaquinone analogues Eur J Med Chem 2011 46 488 496 10.1016/j.ejmech.2010.11.028 21163556

[B16] Pengfei Z Yanxia N Liangqing Y Mo C Congjian X The proliferation, apoptosis, invasion of endothelial-like epithelial ovarian cancer cells induced by hypoxia J Exp Clin Cancer Res 2010 29 124 10.1186/1756-9966-29-124 20831794 PMC2944817

[B17] Deveraux QL Reed JC IAP family proteins–suppressors of apoptosis Genes Dev 1999 1 239 252 9990849 10.1101/gad.13.3.239

[B18] Riccardi C Nicoletti I Analysis of apoptosis by propidium iodide staining and flow cytometry NatProt 2006 1 1458 1461 10.1038/nprot.2006.238 17406435

[B19] Caraglia M Leardi A Corradino S Ciardiello F Budillon A Guarrasi R Bianco AR Tagliaferri P Alpha-Interferon potentiates epidermal growth factor receptor-mediated effects on human epidermoid carcinoma KB cells Int J Cancer 1995 61 342 347 10.1002/ijc.2910610312 7729946

[B20] Xiao-Fen L Cong-Xiang S Zhong Wen Yu-Hong Q Chao-Sheng Y Jun-Qi W Ping-Neng Z Hai-Li W PinX1 regulation of telomerase activity and apoptosis in nasopharyngeal carcinoma cells J Exp Clin Cancer Res 2012 31 12 10.1186/1756-9966-31-12 22316341 PMC3296635

[B21] Lamberti M Porto S Marra M Zappavigna S Grimaldi A Feola D Pesce D Naviglio S Spina AM Sannolo N Caraglia M 5-Fluorouracil induces apoptosis in rat cardiocytes through intracellular oxidative stress J Exp Clin Cancer Res 2012 31 60 (19 July 2012)jmn 10.1186/1756-9966-31-60 22812382 PMC3461434

[B22] Mosmann TJ Rapid colorimetric assay for cellular growth and survival: application to proliferation and cytotoxicity assays Immunol. Methods 1983 65 55 63 10.1016/0022-1759(83)90303-4 6606682

[B23] Rothe G Valet GJ Flow cytometric analysis of respiratory burst activity in phagocytes with hydroethidine and 2′,7′-dichlorofluorescin Leukoc Biol 1990 47 440 448 2159514

[B24] Pourquier P Ueng LM Fertala J Wang D Park HK Essigmann JM Bjornsti MA Pommier Y Induction of reversible complexes between eukaryotic DNA topoisomerase I and DNA-containing oxidative base damages. 7, 8-dihydro-8-oxoguanine and 5-hydroxycytosine Biol Chem 1999 274 8516 8523 10.1074/jbc.274.13.8516 10085084

[B25] Binaschi M Farinosi R Borgnetto ME Capranico G In vivo site specificity and human isoenzyme selectivity of two topoisomerase II-poisoning anthracyclines Cancer Res 2000 60 3770 3776 10919649

[B26] Vitale G Zappavigna S Marra M Dicitore A Meschini S Condello M Arancia G Castiglioni S Maroni P Bendinelli P Piccoletti R Van Koetsveld PM Cavagnini F Budillon A Abbruzzese A Hofland LJ Caraglia M The PPAR-#agonist troglitazone antagonizes survival pathways induced by STAT-3 in recombinant interferon-# treated pancreatic cancer cells Biotechnol Adv 2012 30 1 169 184 10.1016/j.biotechadv.2011.08.001 21871555

[B27] Vitale G Van Eijck CH Van Koetsveld Ing PM Erdmann JI Speel EJ van der Wansem Ing K Mooij DM Colao A Lombardi G Croze E Lamberts SW Hofland LJ Type I interferons in the treatment of pancreatic cancer: mechanisms of action and role of related receptors Ann Surg 2007 246 2 259 268 10.1097/01.sla.0000261460.07110.f2 17667505 PMC1933574

[B28] Perego P Capranico G Supino R Zunino F Topoisomerase I gene expression and cell sensitivity to camptothecin in human cell lines of different tumor types AnticancerDrugs 1994 5 645 649 10.1097/00001813-199412000-00006 7888702

[B29] Gutierrez PL The metabolism of quinone-containing alkylating agents: free radical production and measurement Front Biosci 2000 5 629 638 10.2741/gutier 10877994

[B30] Dandawate PR Vyas AC Padhye SB Singh MW Baruah JB Perspectives on medicinal properties of benzoquinone compounds Mini Rev Med Chem 2010 10 436 454 10.2174/138955710791330909 20370705

[B31] Riedl SJ Renatos M Schwarzenbacher R Zhou Q Sun C Fesik SW Liddington RC Salvesen GS Structural basis for the inhibition of caspase-3 by XIAP Cell 2001 104 791 800 10.1016/S0092-8674(01)00274-4 11257232

